# Supplementary material for: A robust machine learning approach for DC bias prediction in DCO-OFDM based Li-Fi systems
Source: PLoS One. 2025 Nov 10;20(11):e0336234. doi: 10.1371/journal.pone.0336234 (PMC12599948; doi:10.1371/journal.pone.0336234)
Supplement: S1 Appendix — (PDF) [file pone.0336234.s001.pdf]

Table 9. Complete list of RF hyperparameters.

Hyperparameter	Default value	Tuned value
bootstrap	True	True
criterion	squared_error	squared_error (default)
max_depth	None	None
max_features	1.0 (auto)	auto
max_leaf_nodes	None	None (default)
max_samples	None	None (default)
min_impurity_decrease	0.0	0.0 (default)
min_samples_leaf	1	4
min_samples_split	2	2
min_weight_fraction_leaf	0.0	0.0 (default)
n_estimators	100	200
n_jobs	None	None (default)
oob_score	False	False (default)
random_state	None	None (default)
verbose	0	0 (default)
warm_start	False	False (default)
ccp_alpha	0.0	0.0 (default)
max_samples	None	None (default)
