# Supplementary material for: A robust machine learning approach for DC bias prediction in DCO-OFDM based Li-Fi systems
Source: PLoS One. 2025 Nov 10;20(11):e0336234. doi: 10.1371/journal.pone.0336234 (PMC12599948; doi:10.1371/journal.pone.0336234)
Supplement: Attachment — Submitted filename: Reviewer_Comment.docx [file pone.0336234.s002.docx]

The work improves model prediction performance of a previously published work/dataset by using advanced machine learning tools. Their proposed model proves to be more robust, while enhancing accuracy of DC bias prediction compared to previous approaches.

I have reviewed all the concerns and answers to Reviewer #1 and #2. This have been significantly addressed, and should be accepted with the following concerns addressed.

**Concern #1 : Impact on data size**

The authors have given acceptable reasons and advised that the impact of data size on model performance would be conducted as further research. There are some established methods noted by the authors (For example, a statistical measure of reliability such as Kappa index). Adequate citations on this could be included as part of the conclusion section. This was not reflected appropriately in the present manuscript.

**Concern #2 : Further/future studies**

The authors noted in their comments the focus of their paper was on offline data and could be integrated into a real-time LiFi system. This could also be highlighted in the conclusion section as part of future work to include computational resources, latency are critical factors and transition to practical applications.

**Concern #3 : Further/future studies**

The authors noted that ‘the primary objective of work was to enhance prediction accuracy for the dataset originally introduced in Ref [9].’ Considering there was a correlation map for feature analysis, can they authors undertake any other feature analysis technique (permutation feature importance) in enhancing their discussion on feature analysis?

**Concern #4: Manuscript Title**

I believe the work will benefit from writing ML in full (i.e., Machine Learning) in the title as there are already an overkill of abbreviations in the title.

**Concern #5: Clarification of Abstract/Conclusion**

From my understanding of the paper, LPA formed part of the ‘robust’ ML selection process, as it included some statistical testing. This could be clarified in the Conclusion and other sections (Abstract).

**Concern #6: Readability**

Line 406 – 410 is a bit repetitive. Kindly rewrite the identified portion.

**Concern #7: Repeatability/Reproducibility**

For repeatability and reproducibility, hyper-parameter tuning is based on setting the random seed. This could be clarified in Line 406 – 411, where the discussion was given.

**Concern #8: Data Presentation 1**

Fig 7. Can the authors have another sub-figure that plots the actual vs predicted values relative to an ideal fit line? This would quantify departures and deviations in a normalised setting.

**Concern #9: Data Presentation 2**

For table 5, include a column to specify the percentage increase in each stage, relative to [9]

**Concern #10: Data Presentation 3**

In Table 2, keep R2(Mean) values in the same decimal place to enhance presentation.

**Concern #11: Citation**

The statement in line 409 – 411 could benefit from appropriate citations.
