# Supplementary material for: A robust machine learning approach for DC bias prediction in DCO-OFDM based Li-Fi systems
Source: PLoS One. 2025 Nov 10;20(11):e0336234. doi: 10.1371/journal.pone.0336234 (PMC12599948; doi:10.1371/journal.pone.0336234)
Supplement: Attachment — Submitted filename: Response to reviewers r2.docx [file pone.0336234.s003.docx]

Marwah SALMAN

Postgraduate Researcher

Engineering School

University of Leicester

University Road, LE1 7RH, U.K.

Tel: +44(0)7354439156

E-mail: [mtas3@le.ac.uk](mailto:mtas3@le.ac.uk)

Oct 12^th^, 2025.

**Original Manuscript ID:** PONE-D-24-52879 R1

**Original Article Title: “A Robust Machine Learning Approach for DC Bias Prediction in DCO-OFDM Based Li-Fi Systems**”

Dear Prof. Miguel Fernández,

Thank you for allowing a resubmission of our manuscript, with an opportunity to address the reviewers’ comments. We have thoroughly revised the manuscript and addressed all points raised. A detailed, point-by-point response to the comments from Reviewers #1, #2, #3 and #4 are provided below. Our responses are highlighted in blue, while the information added to the manuscript based on the reviewers’ suggestions is highlighted in red.

We are uploading (a) our point-by-point response to the comments under “Author’s Response Files*”*), (b) an updated manuscript with yellow highlighting indicating changes (as “Highlighted PDF*”*), and (c) a clean updated manuscript without highlights (“Main Manuscript”*).*

Regards

Marwah SALMAN (corresponding author)

**Response to Reviewer #1**

Comment 1:

The authors justify the 250-sample dataset by citing prior work and emphasize robustness via shuffling, statistical tests, and future plan for larger dataset. Adequate for a proof-of-concept study, but the limitation should be explicitly noted in the "Limitations" section of the manuscript.

Response:

Thank you for your feedback. We explicitly acknowledged this comment in a new subsection to clearly state that for readers. Therefore, our contribution to Purnita’s work is properly framed.

Limitations section has been added to the revised manuscript, the highlighted PDF on page 17, line 510:

Limitations:

This study acknowledges certain limitations that also present future opportunities for research. Although the dataset used introduces a variety of transmission characteristics and captures key statistical features relevant to DC bias optimization, one limitation is its relatively small size of 250 OFDM symbols. While sufficient for a proof-of-concept demonstration, a larger dataset would further enhance the robustness and generalizability of the results. Future work could focus on incorporating a larger number of training samples while ensuring an even distribution of key parameters. Additionally, the training process could be supported by employing statistical measures such as the Kappa index to validate the adequacy of the chosen dataset size in relation to the model’s performance [48]. Addressing these aspects would further enhance the scalability, reliability, and broader applicability of the proposed methodology.

Comment 2:

Added discussion on physical relevance of features (e.g., BER exclusion due to transmitter-side optimization). Improved but could briefly summarize key features' roles (e.g., how signal statistics directly influence DC bias) in the "Data Description" section.

Response:

Thank you for your feedback. We briefly summarized the influence of the statistical features on the DC bias in the data description section.

The following paragraph has been added to the revised manuscript, the highlighted PDF on page 8, line 259:

The statistical features extracted from each waveform provide a compact representation of the signal and directly inform the DC bias adjustment. Specifically, the minimum and maximum values define the signal’s amplitude range, which determines the margin required to avoid clipping distortion. The mean indicates the average offset of the signal, and the standard deviation characterizes the signal’s power distribution and variability, which influences the scaling factor that primarily controls the required bias. The dataset also records the corresponding BER, serving as a performance benchmark. Together, these features enable the prediction of the optimum DC bias necessary to maintain transmission reliability.

Comment 3:

Acknowledged as future work; proposed block-based approach to reduce computational load. Satisfactory, but a brief computational complexity analysis (e.g., inference time per OFDM block) would strengthen feasibility claims.

Response:

Thank you for your feedback. We have updated the manuscript by adding computational complexity section to briefly explaining that to the readers.

The following section has been added to the revised manuscript, the highlighted PDF on page 13, line 390:

Computational Complexity

In our study, all simulations and model training were performed on Apple MacBook M2 Pro chip (10-core CPU, 16-core GPU, 32 GB RAM). It is important to emphasize that the primary computational burden lies in the offline training phase; once the model is deployed, only inference is required in real-time operation. The selected RF regressor is computationally lightweight at the inference stage, as it only requires the system to provide the key parameters for predicting the optimum DC bias. When the model was tested for a block-based approach, the average inference time was approximately **0.01 ms per OFDM block of 100 symbols**. This latency is negligible compared to the symbol duration in Li-Fi systems, particularly since the update occurs at the block level rather than per individual symbol. Moreover, inference is inherently parallelizable, enabling efficient deployment on embedded processors or FPGA-based systems. These results indicate that the proposed model achieves a favourable balance between accuracy and efficiency, supporting its feasibility for real-time integration in Li-Fi networks.

Comment 4:

Explained parameter ranges and offline training feasibility. Clear, but a table or appendix listing all hyperparameters (default vs. tuned) would enhance reproducibility.

Response:

Thank you for your suggestion. To improve reproducibility, we have added a complete table in the Appendix that lists all Random Forest hyperparameters, including both the tuned values (as reported in Table 4) and those retained at their default settings.

The following table has been added in the Appendix section of the revised manuscript, the highlighted PDF on page 18, line 550:

**Appendix A. Complete Random Forest Hyperparameters**

| Hyperparameter | Default value (scikit-learn) | Tuned value in this study |
| --- | --- | --- |
| bootstrap | True | True |
| criterion | "squared_error" | squared_error (default) |
| max_depth | None | None |
| max_features | 1.0 ("auto") | Auto |
| max_leaf_nodes | None | None (default) |
| max_samples | None | None (default) |
| min_impurity_decrease | 0.0 | 0.0 (default) |
| min_samples_leaf | 1 | 4 |
| min_samples_split | 2 | 2 |
| min_weight_fraction_leaf | 0.0 | 0.0 (default) |
| n_estimators | 100 | 200 |
| n_jobs | None | None (default) |
| oob_score | False | False (default) |
| random_state | None | None (default) |
| verbose | 0 | 0 (default) |
| warm_start | False | False (default) |
| ccp_alpha | 0.0 | 0.0 (default) |
| max_samples | None | None (default) |

Comment 5:

Friedman test used due to non-parametric data; p-values support significance. Well-justified. Consider adding a sentence on effect sizes to complement p-values.

Response:

Thank you for your feedback. We have updated this section to consider complementing p-values with Friedman statistics and effect sizes.

The following paragraph has been added to the revised manuscript, the highlighted PDF on page 11, line 360:

This evaluation compares the performance of the regressors across five iterations of the model training process. Figure 6 presents the Friedman statistics (right axis) alongside the corresponding p-values (left axis) for each iteration. From the second iteration onward, the Friedman test yields p-values below 0.05, indicating statistically significant performance differences among the regressors. To complement the p-values, the effect sizes, expressed as epsilon-squared, ranged from 0.90 to 1.00, reflecting a very strong proportion of variance explained by these differences. Taken together, these results demonstrate that the regressors differ in a statistically significant manner, and that the observed differences are also substantial and practically meaningful.

Comment 6:

Emphasized DCO-OFDM's spectral efficiency but lacked comparison to ACO-OFDM or Flip-OFDM. The authors should add at least one quantitative benchmark (e.g., vs. XGBoost or conventional methods) to strengthen claims of superiority. Without this, the work risks appear incremental.

Response:

We appreciate the reviewer’s suggestion. We would like to clarify that the primary focus of this work is not to compare different OFDM variants (e.g., DCO-OFDM vs. ACO-OFDM or Flip-OFDM) in terms of spectral efficiency, but rather to develop and evaluate a machine learning framework for predicting the DC bias within DCO-OFDM systems. DCO-OFDM outperforms these variants in terms of spectral efficiency and has well-documented advantages in the literature.

In this study, we compared the proposed Random Forest regressor to the polynomial model reported by Purnita et al. [10], which represents the most relevant prior work in DC bias prediction. As detailed in section “RF Model’s Accuracy and Robustness”, our results show that the RF model achieves higher accuracy and robustness compared to the lower performance achieved by the polynomial algorithm. We believe that our work results appear incremental as this comparison demonstrates the superiority of our ML framework in the intended context.

The “RF model’s accuracy and robustness” section has been updated (both title and content) in the revised manuscript. Please see the highlighted PDF on page 15, line 456:

5.4 Superiority of RF Model over Polynomial Regression

In reference [10], data samples were analysed using linear and polynomial regression models to map the features of the OFDM signal to the optimized DC bias. The reported performance metrics were R^2 = 0.8406, RMSE = 0.4279 for linear regression, and R^2 = 0.9677, RMSE = 0.1925 for polynomial regression, suggesting that the polynomial model is the best-performing approach for DC bias prediction in DCO-OFDM systems. To further evaluate the robustness of the polynomial model, we re-assessed its performance on same data but shuffled samples. This resulted in a notable decline in performance, with $R^2$ dropping to 0.8922 and RMSE increasing to 0.3595. In contrast, the RF model developed in this study achieved a consistent R^2 = 0.9450 and RMSE = 0.2603, demonstrating superior accuracy and performance compared to the polynomial. Table [7] summarizes the performance improvement in the RF model relative to the results reported in [10].

The observed performance reduction in the polynomial model appears to rely on the sequential pattern present in the original dataset. This scenario is unlikely to occur in practical transmission where the system parameters dynamically vary in response to changing channel conditions rather than following a predetermined order. The RF model, however, maintained robust performance on both the original and shuffled datasets. As shown in Fig. 7, the proposed RF-based approach provides a meaningful improvement and incremental contribution over prior polynomial model. Thus, highlighting its reliability and suitability for deployment in real-world DCO-OFDM systems.

**Response to Reviewer #2**

Comment 1:

The authors' responses to reviewer comments have been weak, and the revisions made are too light. The paper requires more detailed explanations, important updates, and a stronger, more detailed revision to meet the expected level of quality. The authors need to significantly improve the practical discussion, revise the results, and strengthen the link between the results and the discussion. The methodology chosen has clear weaknesses and requires a more thorough explanation. The paper's technical discussion is weak, and the results are redundant and insufficient, with some illogical comparisons in the figures.

Response:

We thank the reviewer for this observation. While we believe that our initial responses addressed the comments concisely, we acknowledge that some points raised in this comment are broad (e.g., “more detailed explanations,” “important updates,” “a more thorough explanation”), which makes it challenging to identify the specific areas of concern. In the current revision, we have carefully updated the manuscript in line with the reviewers’ feedback to ensure that the explanations are clearer, the revisions are more substantial, and the contributions of our work are better articulated. We hope that these changes sufficiently address this comment.

Comment 2: The literature review is lacking and should be significantly expanded to better position the proposed architecture within existing research. The references are outdated, and newer studies from 2024 must be included to support the arguments made in the paper.

https://doi.org/10.1049/ote2.12111

https://doi.org/10.1007/s11082-024-06692-1

https://doi.org/10.1007/s11082-023-05721-9

https://doi.org/10.1007/s12596-024-01929-4

Response:

We thank the reviewer for highlighting the importance of including up-to-date literature. We would like to clarify that our manuscript does incorporate recent studies: 33 of our cited works are from 2020–2025, 11 from 2015–2019, and only a small number from 2011–2015. This means that approximately 65% of our references are drawn from the most recent six years, ensuring that our work is well-grounded in current advances.

We also note that many of the suggested studies are focused on free-space optical (FSO) communication systems, such as WDM, MIMO-FSO, and OCDMA architectures, typically applied to outdoor, long-range links. These studies are do not directly address our focus, which is the application of machine learning to optimize DC bias in DCO-OFDM-based Li-Fi transmission systems.

Comment 3:

The abstract is unclear and needs to be edited for better clarity. Additionally, the highlights are overly long and should be shortened to a single line for better impact.

Response:

We thank the reviewer for this comment. We respectfully believe that the current abstract is already clear and coherent, as it concisely introduces the problem, explains the motivation, outlines the methodology, and highlights the key findings of our study. We also note that no other reviewers expressed concerns regarding the clarity of the abstract.

Regarding the highlights, we have intentionally included the changes in the response to reviewer file in this revision and structured them to provide concise informative points. We believe the current format provides the necessary clarity and impact.

Comment 4:

The English language quality is inadequate, with grammar, sentence structure, and proofreading needing substantial improvement. The overall writing must be clearer and more professional.

Response:

We thank the reviewer for this feedback. We would like to note that the manuscript has been carefully proofread, including by a native English speaker (the second author). We have undertaken an additional round of thorough language editing to further improve the manuscript. We believe the revised manuscript is now clearer for both native and non-native English readers.

Comment 5:

The evaluation model does not account for varying channel conditions, such as multipath fading and environmental interference. The noise model is limited to additive white Gaussian noise (AWGN), which is not sufficient for this type of work.

Response:

We appreciate the reviewer’s concern regarding channel impairments such as multipath fading and environmental interference. However, we would like to clarify that these effects fall outside the scope of our current study. Our work is not intended to design or evaluate a new communication system; rather, it focuses on improving the machine learning based prediction of DC bias in DCO-OFDM transmission. For this purpose, we employed the dataset provided by Purnita *et al.* [10], which has already been validated and published in the literature. We acknowledge that extending the evaluation to more complex channel conditions is an interesting direction for future research. We mentioned this concern in the revised manuscript in Data description subsection. Please see the highlighted PDF on page 8, line 269:

The simulation model used to generate the dataset considered only an AWGN channel. This choice is justified because AWGN is the dominant noise source in indoor optical wireless communication environments, and the primary objective of DC bias optimization is to mitigate clipping noise at the transmitter before signal propagation. The BER benchmark was therefore selected to ensure clipping noise remained confined to an acceptable level.

**Response to Reviewer #3**

Comment 1: Impact on data size

The authors have given acceptable reasons and advised that the impact of data size on model performance would be conducted as further research. There are some established methods noted by the authors (For example, a statistical measure of reliability such as Kappa index). Adequate citations on this could be included as part of the conclusion section. This was not reflected appropriately in the present manuscript.

Response:

Thank you for your observation. In the previous revision we have reasoned that in the response to reviewer comments only. In this round of revision, we ensured that this information is integrated in the manuscript with adequate citation.

The following statement has been added to the limitation section, the highlighted PDF on page 17, line 518- 524.

Future work could focus on incorporating a larger number of training samples while ensuring an even distribution of key parameters. Additionally, the training process could be supported by employing statistical measures such as the Kappa index to validate the adequacy of the chosen dataset size in relation to the model’s performance [48]. Addressing these aspects would further enhance the scalability, reliability, and broader applicability of the proposed methodology.

Comment 2: Further/future studies

The authors noted in their comments the focus of their paper was on offline data and could be integrated into a real-time Li-Fi system. This could also be highlighted in the conclusion section as part of future work to include computational resources, latency are critical factors and transition to practical applications.

Response:

Thank you for your feedback. In the current revised manuscript, we highlighted these aspects in many sections. We have added computational complexity section and limitation section.

The following section has been added to the revised manuscript, the highlighted PDF on page 13, line 390:

Computational Complexity

In our study, all simulations and model training were performed on Apple MacBook M2 Pro chip (10-core CPU, 16-core GPU, 32 GB RAM). It is important to emphasize that the primary computational burden lies in the offline training phase; once the model is deployed, only inference is required in real-time operation. The selected RF regressor is computationally lightweight at the inference stage, as it only requires the system to provide the key parameters for predicting the optimum DC bias. When the model was tested for a block-based approach, the average inference time was approximately **0.01 ms per OFDM block of 100 symbols**. This latency is negligible compared to the symbol duration in Li-Fi systems, particularly since the update occurs at the block level rather than per individual symbol. Moreover, inference is inherently parallelizable, enabling efficient deployment on embedded processors or FPGA-based systems. These results indicate that the proposed model achieves a favourable balance between accuracy and efficiency, supporting its feasibility for real-time integration in Li-Fi networks.

Limitations section has been added to the revised manuscript, the highlighted PDF on page 17, line 510:

Limitations:

This study acknowledges certain limitations that also present future opportunities for research. Although the dataset used introduces a variety of transmission characteristics and captures key statistical features relevant to DC bias optimization, one limitation is its relatively small size of 250 OFDM symbols. While sufficient for a proof-of-concept demonstration, a larger dataset would further enhance the robustness and generalizability of the results. Future work could focus on incorporating a larger number of training samples while ensuring an even distribution of key parameters. Additionally, the training process could be supported by employing statistical measures such as the Kappa index to validate the adequacy of the chosen dataset size in relation to the model’s performance [48]. Addressing these aspects would further enhance the scalability, reliability, and broader applicability of the proposed methodology.

Comment 3: Further/future studies

The authors noted that ‘the primary objective of work was to enhance prediction accuracy for the dataset originally introduced in Ref [9].’ Considering there was a correlation map for feature analysis, can they authors undertake any other feature analysis technique (permutation feature importance) in enhancing their discussion on feature analysis?

Response:

Thank you for your feedback. Quantitative feature-importance scores (e.g., RF-based ranking) for this dataset have already been reported by Purnita *et al*. [10], as shown in their Table 2. The following statement has been added to the data description section to clarify this point to the reader. Please see the highlighted PDF on page 8, line 274:

However, following Table 2 in Purnita et al., which presents feature-importance scores for this dataset, features with minimal contribution to the DC bias prediction were excluded from our analysis.

Comment 4: Manuscript Title

I believe the work will benefit from writing ML in full (i.e., Machine Learning) in the title as there are already an overkill of abbreviations in the title.

Response:

Thank you for your feedback. We have updated the title from (A robust ML regressor model for predicting the DC bias in DCO-OFDM based Li-Fi technology) to (A Robust Machine Learning Approach for DC Bias Prediction in DCO-OFDM Based Li-Fi Systems).

Comment 5: Clarification of Abstract/Conclusion

From my understanding of the paper, LPA formed part of the ‘robust’ ML selection process, as it included some statistical testing. This could be clarified in the Conclusion and other sections (Abstract).

Response:

Thank you for your feedback. This has been clarified in the abstract and the conclusion sections.

Please see the highlighted PDF on page 1, line 8:

This study builds upon that work by utilizing the same dataset and improving the prediction model with advanced ML tools, such as the LazyPredict algorithm (LPA), to systematically evaluate and select a regression model.

Please see the highlighted PDF on page 18, line 529:

This study employed a robust ML regression algorithm, which incorporates LPA, to evaluate the performance of various ML regression models.

Comment 6: Readability

Line 406 – 410 is a bit repetitive. Kindly rewrite the identified portion.

Response:

Thank you for your observation. We ensured the revised manuscript doesn’t contain any repetitive information, particularly this section of hyperparameter tuning. Please see the highlighted PDF on page 14, lines 437:

The This chosen range of parameters was selected around the bounds of the defaults and typical values known to perform well, while ensuring relevance and computational efficiency. The remaining hyperparameters not included in this table were retained at their default values to maintain practical training without excessive computational cost, as the RF model is known to achieve reliable accuracy with default settings in many applications [45, 46].

Comment 7: Repeatability/Reproducibility

For repeatability and reproducibility, hyper-parameter tuning is based on setting the random seed. This could be clarified in Line 406 – 411, where the discussion was given.

Response:

To improve reproducibility, we have added a complete table in the Appendix that lists all Random Forest hyperparameters, including both the tuned values (as reported in Table 4) and those retained at their default settings.

The following table has been added in the Appendix section of the revised manuscript, the highlighted PDF on page 18, line 550:

**Appendix A. Complete Random Forest Hyperparameters**

| Hyperparameter | Default value (scikit-learn) | Tuned value in this study |
| --- | --- | --- |
| bootstrap | True | True |
| criterion | "squared_error" | squared_error (default) |
| max_depth | None | None |
| max_features | 1.0 ("auto") | Auto |
| max_leaf_nodes | None | None (default) |
| max_samples | None | None (default) |
| min_impurity_decrease | 0.0 | 0.0 (default) |
| min_samples_leaf | 1 | 4 |
| min_samples_split | 2 | 2 |
| min_weight_fraction_leaf | 0.0 | 0.0 (default) |
| n_estimators | 100 | 200 |
| n_jobs | None | None (default) |
| oob_score | False | False (default) |
| random_state | None | None (default) |
| verbose | 0 | 0 (default) |
| warm_start | False | False (default) |
| ccp_alpha | 0.0 | 0.0 (default) |
| max_samples | None | None (default) |

Comment 8: Data Presentation 1

Fig 7. Can the authors have another sub-figure that plots the actual vs predicted values relative to an ideal fit line? This would quantify departures and deviations in a normalised setting.

Response:

Thank you for your valuable suggestion. In the revised manuscript, several changes have been made to improve clarity and presentation. While we have not included an additional plot of actual versus predicted values, we believe that the results are sufficiently and clearly conveyed through the bar charts and the table, which effectively compare the performance of our RF model with the results reported in Purnita’s work.

Comment 9: Data Presentation 2

For table 5, include a column to specify the percentage increase in each stage, relative to [9]

Response:

Thank you for your feedback. We have updated the manuscript to percentage in each stage. Please see the highlighted PDF on page 15, line 456:

5.4 Superiority of RF Model over Polynomial Regression

In reference [10], data samples were analysed using linear and polynomial regression models to map the features of the OFDM signal to the optimized DC bias. The reported performance metrics were R^2^ = 0.8406, RMSE = 0.4279 for linear regression, and R^2^ = 0.9677, RMSE = 0.1925 for polynomial regression, suggesting that the polynomial model is the best-performing approach for DC bias prediction in DCO-OFDM systems. To further evaluate the robustness of the polynomial model, we re-assessed its performance on same data but shuffled samples. This resulted in a notable decline in performance, with R^2^ dropping to 0.8922 and RMSE increasing to 0.3595. In contrast, the RF model developed in this study achieved a consistent R^2^ = 0.9450 and RMSE = 0.2603, demonstrating superior accuracy and performance compared to the polynomial. Table [7] summarizes the performance improvement in the RF model relative to the results reported in [10].

The observed performance reduction in the polynomial model appears to rely on the sequential pattern present in the original dataset. This scenario is unlikely to occur in practical transmission where the system parameters dynamically vary in response to changing channel conditions rather than following a predetermined order. The RF model, however, maintained robust performance on both the original and shuffled datasets. As shown in Fig. 7, the proposed RF-based approach provides a meaningful improvement and incremental contribution over prior polynomial model. Thus, highlighting its reliability and suitability for deployment in real-world DCO-OFDM systems.

Comment 10: Data Presentation 3

In Table 2, keep R2(Mean) values in the same decimal place to enhance presentation.

Response:

Thank you for your feedback. We have updated the table to maintain same decimal places. Please see the highlighted PDF on page 11 and 12.

Comment 10: Citation

The statement in line 409 – 411 could benefit from appropriate citations.

Response:

Thank you for your feedback. We have added appropriate citations to this paragraph. Please see the highlighted PDF on page 14, lines 437:

The This chosen range of parameters was selected around the bounds of the defaults and typical values known to perform well, while ensuring relevance and computational efficiency. The remaining hyperparameters not included in this table were retained at their default values to maintain practical training without excessive computational cost, as the RF model is known to achieve reliable accuracy with default settings in many applications [45, 46].

**Response to Reviewer #4**

Comment 1:

The mathematical description of the IFFT process and DC bias definition lacks sufficient detail. Please expand on how Hermitian symmetry is enforced and explicitly derive the relationship between the scaling factor μ and the DC bias in both linear and dB domains.

Response:

Thank you for your feedback. We expanded on how Hermitian symmetry is enforced in equation (1). For the dB representation of DC bias, this has been given in well-established resources such as books and journal paper. These references provide a detailed mathematical expression of DCO-OFDM transmission and all of them were cited in Subsection (DCO-OFDM fundamentals and clipping noise).

The following paragraphs have been added to the section to expand on Hermitian symmetry. Please see the highlighted PDF on page 4, line 169:

The Hermitian requirements reduce the actual data-transmitting subcarriers to N/2-1. As a result, the complete input vector to the IFFT has the structure:

$$X=[0,X_{1},X_{2}, \ldots, X_{\frac{N}{2}-1}, 0, {X^{*}}_{\frac{N}{2}-1}, \ldots, {X^{*}}_{2}, {X^{*}}_{1}]$$

Please see the highlighted PDF on page 5, line 182:

$$x_{DCO}\left( t \right)= x\left( t \right)+ B_{DC}$$

Where

$$B_{DC}= \mu\sqrt{{E[x(t)}^{2}]}= \mu\sigma_{x}$$

Here, µ represents a positive scaling factor for DC bias adjustment, $\sigma_{x}$ is the signal standard deviation and E[.] is the expectation value [23]. The average electrical power of time domain signal, $P_{elec}$, is proportional to its variance $\sigma_{x}^{2}$. Therefore, the total DC power, $P_{DC}$, can be expressed as:

$$P_{DC}=\left| B_{DC} \right|^{2}+P_{elec}$$

$$P_{DC}={(\mu\sigma_{x})}^{2}+\sigma_{x}^{2}$$

By normalising with respect to $\sigma_{x}^{2}$ [ref], the expression becomes

$$P_{DC norm}=\frac{{(\mu\sigma_{x})}^{2}+\sigma_{x}^{2}}{\sigma_{x}^{2}}= \mu^{2}+1$$

Finally, the normalised DC power in decibels is given by

$$P_{DC norm}\left( dB \right)=10 \log_{10} \left( \mu^{2}+1 \right)$$

Comment 2:

The dataset’s uneven distribution of N (subcarriers) and M (QAM order) may bias the model. Provide summary statistics (histograms or tables) showing how many samples correspond to each (N, M) pair and discuss potential mitigation strategies beyond bootstrap.

Response:

We thank you for your feedback. In the revised manuscript, we now provide summary statistics of the dataset distribution across each (N, M) pair (see Table 1), which highlights the uneven representation of certain parameter combinations. While this imbalance may bias the model, we intentionally retained the dataset in its original form, as the primary goal of this work is to provide improved results over those reported by Purnita *et al.* in [10] using the same dataset. Addressing the imbalance through resampling or dataset modification would alter the basis for comparison and thus falls outside the scope of the present study. The uneven distribution concern has been mentioned in the Limitation section.

The following statement and table have been added to the section to expand on dataset distribution. Please see the highlighted PDF on page 7, line 249:

To illustrate the distribution of samples across different (N, M) combinations, Table [1] summarizes the number of samples available for each case. As shown, the dataset is not evenly distributed, with certain (N, M) pairs being more heavily represented than others.

Table 1. Distribution of samples across subcarriers (N) and modulation order (M)

| N\ M | 4 | 16 | 64 | 256 | 1024 |
| --- | --- | --- | --- | --- | --- |
| 256 | 81 | 35 | 23 | 17 | 6 |
| 512 | 14 | 0 | 0 | 13 | 14 |
| 1024 | 0 | 10 | 0 | 21 | 15 |

Please see the highlighted PDF on page 17, line 510:

Limitations:

This study acknowledges certain limitations that also present future opportunities for research. Although the dataset used introduces a variety of transmission characteristics and captures key statistical features relevant to DC bias optimization, one limitation is its relatively small size of 250 OFDM symbols. While sufficient for a proof-of-concept demonstration, a larger dataset would further enhance the robustness and generalizability of the results. Future work could focus on incorporating a larger number of training samples while ensuring an even distribution of key parameters. Additionally, the training process could be supported by employing statistical measures such as the Kappa index to validate the adequacy of the chosen dataset size in relation to the model’s performance [48]. Addressing these aspects would further enhance the scalability, reliability, and broader applicability of the proposed methodology.

Comment 3:

The work assumes only AWGN noise. Realistic Li-Fi channels often exhibit multipath effects and background light interference. Discuss how these additional impairments could alter the DC bias optimization and whether the model would generalize.

Response:

In our response to Reviewer #1, Concern# 9 in the previous round of revision, given in this comment (The model evaluation does not account for varying channel conditions (e.g., multipath fading, environmental interference). The noise model is limited to AWGN). We mentioned that the BER target for DCO-OFDM simulation in Purnita’s paper was established based on the DC bias optimization process at the transmitter side. This optimization primarily aimed to mitigate the impact of clipping noise, ensuring that the BER remained within the acceptable range before transmission. However, once the signal propagates through the channel, additional noise sources, such as AWGN, multipath fading, and background interference, can further degrade the signal quality. The reason is that their simulation model was limited to AWGN because it is the dominant noise source in indoor optical communication environments. Note that the chosen BER benchmark was specifically designed to confine the clipping impairment at the transmitter to an acceptable level and addressing it separately from any other type of noise in the channel, where the latter can be effectively addressed through channel estimation and equalization techniques at the receiver.

Our study focuses on the ML-driven aspects of the DC optimization process, particularly features analysis, model selection, and performance evaluation, using the dataset in Ref [10]. We mentioned this concern in the revised manuscript in Data description subsection. Please see the highlighted PDF on page 8, line 269:

The simulation model used to generate the dataset considered only an AWGN channel. This choice is justified because AWGN is the dominant noise source in indoor optical wireless communication environments, and the primary objective of DC bias optimization is to mitigate clipping noise at the transmitter before signal propagation. The BER benchmark was therefore selected to ensure clipping noise remained confined to an acceptable level.

Comment 4:

The rationale for eliminating Mean and BER is only briefly justified. Please provide quantitative feature-importance scores (e.g., RF feature ranking) and detail any correlations among the retained features to demonstrate the robustness of feature selection.

Response:

We thank you for this comment. Quantitative feature-importance scores (e.g., RF-based ranking) for this dataset have already been reported by Purnita et al. [10], as shown in their Table 2. In particular, the features Mean and BER were found to contribute minimally compared with others such as Max, std, and M. To avoid redundancy, the feature selection process in this study therefore follows Purnita et al., and our results demonstrate that even with this reduced feature set, the proposed approach achieves improved prediction performance.

The following statement has been added to the data description section to clarify this point to the reader. Please see the highlighted PDF on page 8, line 274:

However, following Table 2 in Purnita et al., which presents feature-importance scores for this dataset, features with minimal contribution to the DC bias prediction were excluded from our analysis.

The following statement has been updated in the “model improvement based on feature selection and hyperparameter tuning” section to clarify this point to the reader. Please see the highlighted PDF on page 14, line 413:

As a result, the Mean and BER features are eliminated due to their weak direct and indirect correlations with the DC bias. The mean value exhibits a weak correlation primarily because the OFDM signal inherently follows a Gaussian distribution with a zero mean. In such a distribution, the overall average of OFDM signal amplitudes is symmetrically distributed around zero regardless of the DC bias level. Consequently, variations in the DC bias do not significantly alter the mean value of the OFDM signal, leading to a weak or negligible correlation between them. Meanwhile, the BER is excluded because the DC bias primarily affects the transmitted signal on the transmitter side of the system, while BER is determined on the receiver side after performing the demodulation process. As a result, including BER in the prediction process would not be practical or meaningful in this context. This procedure of feature refinement is particularly valuable for understanding the impact of each feature on DC bias, which is critical in practical applications. Following this feature selection process, the RF model achieved R^2^ = 0.9436 and RMSE= 0.2635. The results show a slight decrease in performance compared to the initial features. However, training the model with the most relevant and correlated features improves its generalization and practicability in deployment.

Comment 5:

The choice of a single 70/30 split repeated with shuffling is less standard than k-fold cross-validation. Compare the train–test approach with k-fold (e.g., 5-fold) CV to quantify any overfitting risk and to validate the reported performance.

Response:

Thank you for your recommendation. We opted for a train-test split rather than cross-validation to ensure a direct and fair comparison with the results in Purnita *et al.* [10]. To mitigate the risk of overfitting, we employed several techniques, including data normalization, multiple training iterations, statistical validation, and hyperparameter tuning. We believe that these measures collectively enhance the robustness and generalization capability of our ML model.

Comment 6

While the Friedman test is appropriate, please report the test statistic, degrees of freedom, and exact p-values. Also, consider a post-hoc test (e.g., Nemenyi) to identify which regressors differ significantly.

Response

We thank the reviewer for this suggestion. In the revised manuscript, we now report the exact p-values for each iteration of the Friedman test, which are 0.01 from the second iteration, indicating statistically significant differences among the regressors. We also modified Figure 6 to clearly present the Friedman statistics alongside the corresponding p-values. We complemented the p-values with effect sizes which highlights the strong practical significance of these differences. Although degrees of freedom and a post-hoc test were not considered, we believe the reported results are sufficient to demonstrate that the differences are also practically meaningful.

The following paragraph has been added to the revised manuscript. Please see the highlighted PDF on page 11, line 360:

This evaluation compared the performance of the regressors across five iterations of the model training process. Fig. 4 presents the Friedman statistics (right axis) alongside the corresponding p-values (left axis) for each iteration. From the second iteration, the Friedman test yields p-values of 0.01, indicating statistically significant performance differences among the regressors. To complement the p-values, the effect sizes, expressed as epsilon-squared, ranged from 0.90 to 1.00, reflecting a very strong proportion of variance explained by these differences. Taken together, these results demonstrate that the regressors differ in a statistically significant manner, and that the observed differences are also substantial and practically meaningful.

Comment 7:

The grid search explored only six RF hyperparameters. Explain why other important parameters (e.g., max_depth, min_impurity_decrease) were kept at defaults, and discuss the computational cost versus performance trade-offs.

Response:

Thank you for your feedback. In our study, the grid search was focused on six hyperparameters that have the most significant influence on Random Forest performance: bootstrap, max_features, min_samples_leaf, min_samples_split, and n_estimators. Other hyperparameters (e.g. max_depth and min_impurity_decrease) were left at their default values for two main reasons:

1. Computational cost: The search space grows exponentially as additional hyperparameters are included. Expanding the grid to cover all parameters would have substantially increased training time without proportionate performance gains.
2. Performance trade-offs: Prior studies and our preliminary experiments indicated that Random Forest models often achieve strong performance with default values.

With the selected six tuned parameters, our model achieved an R² of 0.9450 and an RMSE of 0.2603, indicating robust predictive accuracy. Given this level of performance, further exhaustive tuning of additional parameters would have offered only marginal improvements at a disproportionately higher computational cost.

The following paragraph has been added to the revised manuscript. Please see the highlighted PDF on page 14, lines 437:

This chosen range of parameters was selected around the bounds of the defaults and typical values known to perform well, while ensuring relevance and computational efficiency. The remaining hyperparameters not included in this table were retained at their default values to maintain practical training without excessive computational cost, as RF model often achieves strong performance with default settings [45, 46]. To ensure that the experimental setup can be fully replicated, a complete table in Appendix A lists all the hyperparameters, including both the reported tuned values and those retained at their default.

After tuning the hyperparameters with the selected input features, the model performance improved, with R^2^ increasing to 0.9450 and RMSE decreasing to 0.2603, as shown in Table 6. Although this represents only a modest improvement, it constitutes an important and necessary step. The DC bias is strongly correlated with the BER feature, which accounts for the slightly higher performance when BER is included. As mentioned earlier in this section, in practical transmitter-side DC bias optimization, incorporating BER as a predictor is not feasible. Excluding it, therefore, ensures that the model delivers strong and reliable performance suitable for real-world deployment.

Comment 8

Furthermore, the work would greatly benefit from including and referencing more recent material on practical implementations of machine learning methods across several domains. It is recommended to engage in discussions on the comparison of results and the integration of these concepts into your work. You may find more information on this topic in the following articles:

<https://doi.org/10.1007/s11004-023-10116-3>,

<https://doi.org/10.1007/s11069-023-06322-1>,

<https://doi.org/10.1007/s12583-021-1525-9>,

<https://doi.org/10.1007/s12583-021-1407-1>.

Response

We thank the reviewer for this suggestion. While our primary focus is on ML applications in wireless communications, we have carefully reviewed the recommended articles and integrated them into the manuscript where appropriate, as follow:

- The study “Toward the reliable prediction of reservoir landslide displacement using earthworm optimization algorithm-optimized support vector regression (EOA-SVR)” was cited in relation to the Friedman test analysis.
- The work “Landslide Susceptibility Mapping in Terms of the Slope-Unit or Raster-Unit, Which is Better?” was referred to in the context of the p-value reference for statistical significance.
- The paper “A Comparative Study of Supervised Classification Methods for Investigating Landslide Evolution in the Mianyuan River Basin, China” was included to highlight the accuracy of Random Forest in many applications when using default hyperparameter settings.
- The article “Automated Machine Learning-Based Landslide Susceptibility Mapping for the Three Gorges Reservoir Area, China” was referenced to emphasize the wide applicability of ML methods across diverse domains beyond communications.

We also highlighted the new added references to the manuscript in the highlighted version. Please see references 38, 37 ,46, and 8.

Comment 9:

Some figures (e.g., bar charts of R² and RMSE) lack error bars or confidence intervals. Add them to convey statistical variance. Ensure all figures are integrated into the text at appropriate points and are high resolution.

Response

We thank the reviewer for this feedback. We have updated the bar charts of R² and RMSE to include error bars. Regarding figure integration, we note that PLOS ONE requires figures to be submitted as separate files rather than embedded in the manuscript during the initial submission. Following this guideline, each figure has been uploaded as an individual high-resolution file (e.g., Fig1.pdf), and the corresponding figure captions have been inserted into the manuscript text immediately after the first paragraph where each figure is cited.

However, for ease of review, we have maintained the figures integrated within the highlighted version of the manuscript to make it easier for you to track the changes. Please find the Latex template in this link <https://journals.plos.org/plosone/s/latex>.

Comment 10:

The manuscript still contains grammatical errors and inconsistencies (e.g., plural/singular agreement, verb tense). A thorough language edit is needed. Additionally, ensure all sections are numbered and citations follow the journal’s prescribed style.

Response

We thank the reviewer for this feedback. The manuscript has been thoroughly revised to correct grammatical errors. Although the journal’s LaTeX and Word templates format sections without numbering, we have added section numbers in response to feedback from multiple reviewers. All citations have been checked and formatted according to the journal’s prescribed style. Please find the Latex template in this link <https://journals.plos.org/plosone/s/latex>.
